# The effects of platelet activating factor and retinoic acid on the expression of ELAM-1 and ICAM-1 and the functions of neutrophils

**DOI:** 10.1155/S0962935195000172

**Published:** 1995-03

**Authors:** Si-Feng Chen

**Affiliations:** 1 Department of Pathophysiology Second Military Medical University 800 Xiang Yin Road Shanghai 200433 China

## Abstract

Preincubation of pulmonary microvascular endothelial cells (PMVECs) with platelet-activating factor (PAF) for 3.5 h increased the adhesion rate of polymorphonuclear leukocytes (PMNs) to PMVECs from 57.3% to 72.8% (p < 0.01). Preincubation of PMNs with PAF also increased PMN-PMVEC adhesion rate. All-trans retinoic acid (RA) blocked the adherence of untreated PMNs to PAF-pretreated PMVECs but not the adherence of PAF-pretreated PMNs to untreated PMVECs. PAF increased the expression of intercellular adhesion molecule-1 (ICAM-1) and E-selection (ELAM-1) on PMVECs, PMN chemotaxis to zymosan-activated serum and histamine, and PMN aggregation and the release of acid phosphatase from PMNs. Co-incubation of RA inhibited PAF-induced PMN aggregation, the release of acid phosphatase from PMNs, and PMN chemotaxis to zymosan-activated serum and histamine while the expression of ICAM-1 and ELAM-1 did not change. Our results suggest that RA can be used to ameliorate PMN-mediated inflammation.


Research Paper

Mediators of Inflammation 4, 103–106 (1995)

### The effects of platelet activating factor and retinoic acid on the expression of ELAM-1 and ICAM-1 and the functions of neutrophils

**Si-Feng Chen**

Department of Pathophysiology, Second Military Medical University, 800 Xiang Yin Road, Shanghai 200433, Peoples' Republic of China

CA Corresponding Author

**Preincubation of pulmonary microvascular endothelial cells (PMVECs) with platelet-activating factor (PAF) for 3.5 h increased the adhesion rate of polymorphonuclear leukocytes (PMNs) to PMVECs from 57.3% to 72.8% ($p$ < 0.01). Preincubation of PMNs with PAF also increased PMN–PMVEC adhesion rate. All-trans retinoic acid (RA) blocked the adherence of untreated PMNs to PAF-pretreated PMVECs but not the adherence of PAF-pretreated PMNs to untreated PMVECs. PAF increased the expression of intercellular adhesion molecule-1 (ICAM-1) and E-selection (ELAM-1) on PMVECs, PMN chemotaxis to zymosan-activated serum and histamine, and PMN aggregation and the release of acid phosphatase from PMNs. Co-incubation of RA inhibited PAF-induced PMN aggregation, the release of acid phosphatase from PMNs, and PMN chemotaxis to zymosan-activated serum and histamine while the expression of ICAM-1 and ELAM-1 did not change. Our results suggest that RA can be used to ameliorate PMN-mediated inflammation.**

**Key words**: Cellular adhesion, Cellular adhesion molecules, Chemotaxis, Neutrophils, Platelet-activating factor, Retinoids, Vascular endothelium

### Introduction

Polymorphonuclear neutrophils (PMNs) play a key role in the inflammatory response underlying many diseases. PMNs are capable of chemotaxis, phagocytosis, oxygen-free radical production, and degranulation in response to a variety of stimuli. Overstimulation of PMNs can result in host tissue injury. Inhibition of PMN functions may be beneficial to inflammatory diseases such as systemic inflammatory reaction syndrome.[1] During inflammation, leukocytes may adhere firmly in microvascular endothelial cells (ECs) whereupon they project pseudopodia and migrate across endothelial monolayers into traumatized interstitia through diapedesis. The localized adhesion of leukocytes to ECs is mediated at least partly by adhesion molecules on leukocytes and their counterpart molecules on ECs such as intercellular adhesion molecule-1 (ICAM-1) and E-selectin (ELAM-1).[2]

All-trans retinoic acid (RA) has beneficial effects when used in a variety of inflammatory skin conditions.[3] Retinoids significantly inhibit the migration of neutrophils from the blood into tissues in volunteers.[4] This study observed the effects of PAF and RA on the functions of polymorphonuclear neutrophil leukocytes (PMNs) and the adhesion of PMNs to pulmonary microvascular endothelial cells (PMVECs).

### Materials and Methods

*Reagents:* Monoclonal antibodies 1.2B6 (anti-human ELAM-1) and 6.5 (anti-human ICAM-1) were gifts of D. O. Haskard, Royal Postgraduate Medical School, University of London. All-trans retinoic acid, Dulbecco's Modified Eagle Medium (DMEM) and dextran T500 were purchased from Sigma. Other reagents were purchased from Shanghai Chemical Reagent Co.

*Zymosan activated serum:* Zymosan A was suspended in normal rat serum at a concentration of 2 mg/ml and incubated at 37°C for 30 min. Then the serum was centrifuged at 1500 × **g** for 10 min. The supernatant was divided into aliquots and kept frozen at –20°C until use.

*Isolation of endothelial cells:* Sprague–Dawley rats weighing 80–100 g were anaesthetized with urethane (2 g/kg body weight) and heparinized (1000 units per animal) intraperitoneally. The animals were exsanguinated by cutting bilateral carotid arteries. The blood remaining in the pulmonary vascular bed was washed out with Hanks' solution. The lungs were isolated. The tissues of the lung surface or edge were cut into separate pieces of 1 × 1 × 1.5 mm dimensions. Ten pieces were placed in a flask with a 45 $cm^2$ bottom surface and cultured in DMEM supplemented with 20% foetal bovine serum. No

© 1995 Rapid Communications of Oxford Ltd antibiotics, growth factors or extracellular matrix proteins were added. After 60 h culture, the tissues were discarded and the medium partially changed. The flask contained only ECs and blood cells. The cells were subcultured with 0.08% trypsin in Hank's solution without calcium between days 6 and 10. The cells were identified as pulmonary PMVECs according to morphological and functional criteria.[5]

*Measurement of PMN–PMVEC adherence:* PMNs were isolated from heparinized rat blood with dextran sedimentation and centrifugation on Ficoll–Hypaque discontinuous density as reported previously.[6] Cell viability determined by the Trypan Blue exclusion test was more than 99%. To reduce the variations in the experiment, the adherence of PMNs to PMVECs in 96-well plates was measured by the following two methods. PMNs ($1.0 \times 10^5$/well) in Hanks' solution were added to PMVEC monolayers ($4 \times 10^4$ cells/well) pretreated as in the experimental protocol. After incubation at 37°C in 5% $CO_2$ and 95% air for 30 min, PMVEC monolayers with adherent PMNs were washed gently with the culture medium. First, the number of adhered PMNs was calculated from the counting difference between PMNs added and aspirated (total cell count of the aspirated washing medium). Secondly, the number of adhered PMNs was calculated by using phase-contrast microscopy. The total number of adhered PMNs = (the area of one culture well/the area of one field of view) × the number of adhered PMNs of one field of view. The results were expressed as the percentage increase compared with the control. The results from the two methods were not significantly different. The average data obtained by the two methods was presented in the results.

*Determination of the expression of ICAM-1 and ELAM-1 on PMVECs by using ELISA:* PMVECs were plated in 96-well microtitre plates at a concentration of $4 \times 10^4$ cells and were preincubated with culture media alone, PAF ($10^{-8}$ mol/1) and PAF plus RA ($4 \times 10^{-9}$ mol/1) for 4 h at 37°C. Culture supernatant (100 μl) containing monoclonal antibodies 1.2B6 or 6.5B% was added. The plates were incubated at 37°C for 30 min. After washing, 100 μl peroxidase-conjugated goat anti-mouse IgG, diluted 1:500, was added to each well and the plates were incubated for 30 min. The plates were washed again. *o*-Phenylenediamine (100 μl) and 30 μg $H_2O_2$ in 100 μl citrate–phosphate buffer (pH 5.0) were added. The plates were incubated at 37°C for 30 min. The chromogenic reaction was stopped with 100 μl 2N $H_2SO_4$ and the plates read spectrophotometrically at 492 nm on an ELISA reader (DG 3022A, East China Electron Tube Factory).

*The effects of RA on PMN–PMVEC adherence:* The ECs were cultured to a confluent monolayer on 96-well plates and preincubated for 3.5 h with culture media alone, PAF ($10^{-8}$ mol/l) and RA ($4 \times 10^{-9}$ mol/l) plus PAF. All the media used were adjusted to contain 0.1% alcohol to prevent precipitation of RA. PMNs ($1 \times 10^5$ cells/well) were added and incubated for 30 min. The adhesion rate was measured as described elsewhere. PMNs were preincubated in the same way as that used for PMVECs. After preincubation, PMNs were added to untreated PMVECs and the adhesion rate was measured.

*Chemotaxis assay:* PMN chemotaxis was measured as describe by Nelson.[7] PMNs were suspended in culture medium containing RA (0, $10^{-8}$ and $10^{-10}$ mol/l). PMNs at a concentration of $1 \times 10^5$ cells/well were added to agarose holes (ten holes in each group) and incubated at 37°C in 5% $CO_2$ for 18 h. The cells were dehydrated in 75% ethanol and stained by Wright's method. The distance of the migration front towards the wells containing either zymosan-activated serum (ZAS) or histamine ($8.7 \times 10^{-8}$ mol/l) were measured with an internal microscope micrometer.

*PMN aggregation:* PMNs were preincubated with RA ($1.8 \times 10^{-9}$ mol/l) or medium alone for 4 h. The PMN aggregation caused by PAF was determined on a PPP automatically balanced platelet aggregator (Shanghai Keda Apparatus Factory). PAF (50 μl, $10^{-8}$ mol/l) caused maximal aggregation. PAF (30 μl, $10^{-8}$ mol/l) was used to measure the samples. PMN aggregation (%) = PMN aggregation of sample × 100%/maximal PMN aggregation.

*The release of acid phosphatase from PMNs:* PMNs were incubated with control medium, PAF ($10^{-8}$ mol/l) alone or PAF ($10^{-8}$ mol/l) plus RA ($10^{-11}$ to $10^{-8}$ mol/l) for 4 h with ten samples in each group. The PMNs were then centrifuged and acid phosphatase activity of the supernatant was measured by using a colorimetric method.

*Statistical analyses:* Data are expressed as the mean ± S.E.M. Statistical analyses were performed by unpaired Student's *t*-tests.

### Results

*The effects of RA on PMN–PMVEC adherence:* Stimulation of PMVEC with PAF for 3.5 h increased PMN–PMVEC adhesion rate by 27%. In the presence of RA, the ability of PAF to increase adhesion rate decreased significantly (Fig. 1). Preincubation of PMNs with PAF also increased PMN–PMVEC adhesion rate significantly ($p < 0.01$). RA did not block the adhesion of PAF-pretreated PMNs to PMVECs (Fig. 1).

*Expression of ELAM-1 and ICAM-1 on PMVECs:* PAF increased the expression of both ELAM-1 and ICAM-

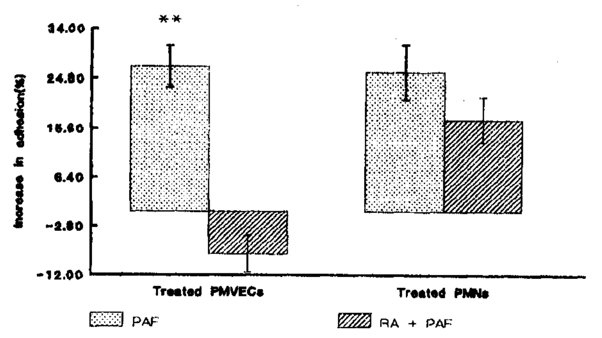

FIG. 1. The effects of RA on the adherence of untreated PMNs to PAF-treated PMVECs (treated PMVECs) and the adherence of PAF-treated PMNs to untreated PMVECs (treated PMNs). $n = 8$. $^{**}p < 0.01$ *vs* RA.

1, whereas RA did not influence the expression of these two molecules (Fig. 2).

*PMN chemotaxis:* Chemotaxis distances of control PMNs to ZAS and histamine were 344.2 ± 6.0 μM and 270.0 ± 7.34 μM, respectively. RA decreased ZAS or histamine-induced PMN chemotaxis (Table 1).

*PMN aggregation:* RA decreased PAF-induced PMN aggregation from 73.0 ± 2.5% to 57.5 ± 3.4% ($p < 0.01$).

*The release of acid phosphatase from PMNs:* PAF increased the release of acid phosphatase significantly whereas RA ($10^{-12}$ to $10^{-8}$ mol/l) did not affect the release of acid phosphatase.

### Discussion

Acute inflammatory reactions are characterized by the local accumulation of leukocytes at the inflammatory sites. A major target of inflammatory mediators is endothelial cells, which *in vitro* may express several mediator-inducible cell-surface molecules that bind leukocytes through specific ligand interaction.[8] ELAM-1 and ICAM-1 mediate the attachment of PMNs to endothelial cells.

PAF is one of the major mediators of inflammatory reactions, such as those elicited by liposaccharide.

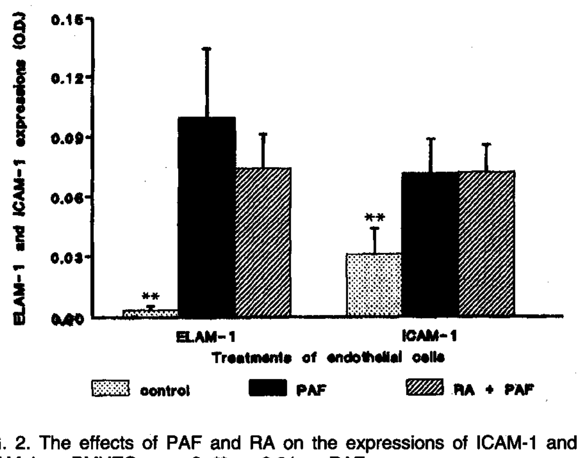

FIG. 2. The effects of PAF and RA on the expressions of ICAM-1 and ELAM-1 on PMVECs. $n = 8$. $^{**}p < 0.01$ *vs* PAF.

**Table 1.** The effect of retinoic acid on PMN chemotaxis to zymosan-activated serum and histamine

| Retinoic acid concentration (mol/l) | Chemotaxis distance (μM) | |
|---|---|---|
| | Zymosan-activated serum | Histamine |
| 0 (control) | 270.0 ± 7.3 | 344.2 ± 6.0 |
| $10^{-9}$ | 253.8 ± 7.9** | 302.0 ± 7.1** |
| $10^{-8}$ | 191.3 ± 3.3** | 265.5 ± 4.7** |

$^{**}p < 0.01$ *vs* control. $n = 10$.

PAF increases PMN adhesion and degranulation.[8–10] PAF may be a common messenger signalling the increase of leukocyte adherence to endothelial cells elicited by a group of mediators, such as thrombin, leukotriene $C_4$ and $D_4$, $H_2O_2$ and interleukin-2.[11–13] The present study showed that PAF increased PMN aggregation, PMN–PMVEC adhesion, the release of acid phosphatase from PMNs, and the expressions of ELAM-1 and ICAM-1 on PMVECs. PAF-induced PMN–EC (endothelial cell) adhesion was both protein synthesis dependent and independent.[9] PAF increased CD18 expression on PMNs.[14] Hattori *et al.* reported that intracellular granule components GMP-140 in ECs might translocate to the cell surface rapidly when ECs were activated by histamine, thrombin, phorbol myristate acetate or A23187.[15] Whether GMP-140 takes part in the process of PAF-induced PMN–EC adhesion is unknown. PAF is a phospholipid and may insert into the membrane lipid bilayer. Washing EC monolayers after PAF treatment did not decrease PMN adherence.[9] PAF may stimulate the production and the release of other mediators. Many PAF-induced mediators may increase PMN–EC adhesion. In conclusion, four possible mechanisms exist in PAF-induced PMN–EC adhesion: (1) the expression of ELAM-1 and ICAM-1 on ECs, and CD18 on PMNs; (2) the translocation of GMP-140 in ECs; (3) the interaction of EC-bound PAF with PAF receptors on PMN membranes; and (4) the actions of PAF-induced mediators.

Endothelial cells express a retinoic acid receptor.[16] RA decreased scalding- and platelet-activating factor-induced pad oedema and high vascular permeability of rats (authors' unpublished data). Retinoids ameliorated the injury in rat lung and skin sites after treatment with bovine serum albumin and antibodies to bovine serum albumin.[17] Twenty-four hours following RA treatment, endothelial cells occupied a greater area than control.[18] Retinoids inhibited proliferation of endothelial cells from skin and aorta,[19] but it is reported that RA enhanced the mitogenic effect of epidermal growth factor on cultured bovine corneal endothelial cells.[20] Thus, retinoids may play a role in the regulation of endothelial cell function. In this experiment, it is found that although RA decreased PAF-induced adherence of PMNs to PAF-pretreated PMVECs, the expressions of ELAM-1 and ICAM-1 on PMVECs did not change significantly. The inhibition of PMN–PMVEC adhesion by RA may not be due to the decrease in the expression of ICAM-1 and ELAM-1.

Since RA inhibited the adherence of fresh PMNs to PAF-pretreated PMVECs but not PAF-pretreated PMNs to untreated PMVECs, RA probably inhibited PMN–PMVEC adhesion by affecting PMVEC reactivity.

Retinoids inhibited the migration of PMNs from the blood to tissues.[4] RA inhibited superoxide anion production, proteolytic enzyme and arachidonic acid release from PMNs.[17,21] Robinson reported that all-trans-retinal stimulated $O_2$ release but not granule exocytosis.[22] Retinoids inhibited tumour necrosis factor and nitric oxide production of murine peritoneal macrophages,[23] and interleukin-1-induced cytokine synthesis in human monocytes and lung fibroblasts.[24,25] RA treatment inhibited degranulation of extracellular matrix and type IV collagen by 50 to 60%.[26] Most of the mediators reduced by retinoids are pro-inflammatory mediators. In this experiment, RA inhibited PMN adhesion, aggregation and chemotaxis. Thus, retinoids may be a group of effective anti-inflammatory compounds.

### References

1. Bone RC, Balk RA, Fein AM, Knaus WA, Cerra TB. Definitions for sepsis and organ failure and guidelines for the use of innovative therapies in sepsis. *Chest* 1992; **101**: 1644–1655.
2. Bricoe DM, Cotran RS, Pober JS. Effects of tumor necrosis factor, lipopolysaccharide, and IL-4 on the expression of vascular cell adhesion molecule-1 *in vivo*: correlation with CD3+ T cell infiltration. *J Immunol* 1992; **149**: 2954–2960.
3. Boyd AS. An overview of retinoids. *Am J Med* 1989; **86**: 568–574.
4. Dubertret L, Lebreton C. Functions of neutrophils *in vivo* on the human skin. Applications to the study of psoriasis and anti-inflammatory agents. *Pathol Biol, Paris* 1987; **35**: 1389–1394.
5. Chen SF, Li SH. A new method for isolation of microvascular endothelial cells avoiding biochemical and mechanical injuries. *Microvas Res* 1995 (in press).
6. Zimmerman GA, McIntyre TM, Prescott SA. Thrombin stimulates the adherence of neutrophils to human endothelial cells *in vitro*. *J Clin Invest* 1985; **76**: 2235–2246.
7. Nelson RD, Quie PG, Simmons RL. Chemotaxis under agarose: a new and simple method for measuring chemotaxis and spontaneous migration of human polymorphonuclear leukocytes and monocytes. *J Immunol* 1975; **115**: 1150–1156.
8. Kuijper TW, Hakkert BC, Hoogerwerf M, Leeuwenberg JF, Ross D. Role of endothelial leukocyte adhesion molecule-1 and platelet activating factor in neutrophil adherence to IL-1-prestimulated endothelial cells: endothelial leukocyte adhesion molecule-1-mediated CD18 activation. *J Immunol* 1991; **147**: 1369–1376.
9. Ding ZL, Li SH, Wu ZL. Mechanisms of endothelial cell-dependent leukocyte adhesion stimulated by platelet-activating factor. *Inflammation* 1992; **16**: 179–186.
10. Zimmerman GA, McIntyre TM. Neutrophil adherence to human endothelium *in vitro* occurs by CDw18 (Mol, MAC-1/LFA-1/GP150,95) glycoprotein-dependent and independent mechanisms. *J Clin Invest* **81**: 531–537.
11. Zimmerman GA, McIntyre TM, Mehra M, Prescott SM. Endothelial cell-associated platelet-activating factor: a novel mechanism for signaling intercellular adhesion. *J Cell Biol* 1990; **110**: 529–540.
12. McIntyre TM, Zimmerman GA, Prescott SM. Leukotrienes $C_4$ and $D_4$ stimulate human endothelial cells to synthesize platelet activating factor and bind neutrophils. *Proc Natl Acad Sci USA* 1986; **83**: 1557–1562.
13. Toothil VJ, Van Mourik JA, Niewenhuis HK, Metzelaar MJ, Pearson JD. Characterization of the enhanced adhesion of neutrophil leukocytes to thrombin-stimulated endothelial cells. *J Immunol* 1990; **145**: 283–291.
14. Kubes P, Suzuki M, Granger DN. Modulation of PAF-induced leukocyte adherence and increased microvascular permeability. *Am J Physiol* 1990; **259**: G859–G864.
15. Hattori R, Hamilton KK, Fugate RD. Stimulated secretion of endothelial von Willebrand factor is accompanied by rapid redistribution to the cell surface of the intracellular granule membrane protein GMP-140. *J Biol Chem* 1989; **264**: 7768–7771.
16. Fesus L, Nagy L, Basilion JP, Davies PJ. Retinoic acid receptor transcripts in human umbilical vein endothelial cells. *Biochem Biophys Res Commun* 1991; **179**: 32–38.
17. Varani J, Jones J, Dame M, Sulavik C, Gibbs DF, Johnson KJ. Effects of all-trans retinoic acid on neutrophil-mediated endothelial cell injury *in vitro* and immune complex injury in rats. *Am J Pathol* 1991; **139**: 901–909.
18. Braunhut SJ, Palomares M. Modulation of endothelial cell shape and growth by retinoids. *Microvasc Res* 1991; **41**: 47–62.
19. Imcke E, Ruszczak Z, Mayer-da-Silva A, Detmar M, Orfanos CE. Cultivation of human dermal microvascular endothelial cells *in vitro*: immunocytochemical and ultrastructural characterization and effect of treatment with three synthetic retinoids. *Arch Dermatol Res* 1991; **283**: 149–157.
20. Bonne C, Junquero D, Driot JY, Coquelet C, Modat G. Effect of retinoids on the growth of corneal endothelial cells in culture. *Opthalmologie* 1990; **4**: 84–87.
21. Nigam S. Inhibition of lipoxygenase products by retinoids in human blood cells. *Dermatologica* 1987; **175**(suppl 1): 73–80.
22. Robinson JM, Badwey JA, Karnovsky ML. Cell surface dynamics of neutrophils stimulated with phorbol esters or retinoids. *J Cell Biol* 1987; **105**: 417–426.
23. Mehta K, McQueen T, Tucker S, Pandita R, Aggarwal BB. Inhibition by all-trans-retinoic acid of tumor necrosis factor and nitric oxide production by peritoneal macrophages. *J Leukoc Biol* 1994; **55**: 336–342.
24. Gross V, Villiger PM, Zhang B, Lotz M. Retinoic acid inhibits interleukin-1-induced cytokine synthesis in human monocytes. *J Leukoc Biol* 1993; **54**: 125–132.
25. Zitnik RJ, Kotloff RM, Latifpour J, Zheng T, Whiting NL, Schwalb J. Retinoic acid inhibition of IL-1-induced IL-6 production by human lung fibroblasts. *J Immunol* 1994; **152**: 1419–1427.
26. Nakajima M, Lotan D, Baig MM, Carralero RM, Wood WR, Hendrix MJ, Lotan R. Inhibition by retinoic acid of type IV collagenolysis and invasion through reconstituted basement membrane by metastatic rat mammary adenocarcinoma cells. *Cancer Res* 1989; **49**: 1698–1706.

ACKNOWLEDGEMENT. This project was supported by the National Natural Scientific Foundation of China (no. 39970780).

**Received 15 September 1994; accepted in revised form 1 December 1994**
